# P-1411. Latent Tuberculosis Infection Screening in Patients Living with Human Immunodeficiency Virus

**DOI:** 10.1093/ofid/ofaf695.1598

**Published:** 2026-01-11

**Authors:** Seung Ah Kang, Crystal Zheng

**Affiliations:** Tulane University, New Orleans, LA; Tulane University, New Orleans, LA

## Abstract

**Background:**

People living with HIV (PLHIV) are at higher risk of progression from latent tuberculosis infection (LTBI) to active tuberculosis (TB). Guidelines from the U.S. Department of Health and Human Services recommend LTBI screening at HIV diagnosis and after immune recovery, with annual screening only for those with risk factors for TB. Despite this, routine annual screening may still occur in clinical practice. Data on the positive predictive value of interferon-gamma release assays (IGRAs) for LTBI screening in PLHIV, particularly living in low-incidence countries, is limited, and testing in these settings may lead to false positives. We evaluated the appropriateness of LTBI screening according to recommended guidelines at an HIV Clinic in New Orleans.

**Methods:**

We conducted a retrospective chart review of PLHIV who underwent LTBI screening with IGRAs at an HIV clinic in New Orleans from January 1, 2022, to December 23, 2023. For those with positive or invalid/indeterminate tests, we obtained data on demographics, CD4 count, history of LTBI or active TB, and risk factors for TB. LTBI screening was considered appropriate if there was no prior LTBI or active TB diagnosis and if the patient met one or more of the following: IGRA testing at the time of HIV diagnosis (or upon establishing care at the clinic), CD4 count recovery above 200 cells/µL, or the presence of ongoing TB risk factors.

**Results:**

During the study period, there were 6,222 visits, involving a total of 1,555 patients. Of these, 1,258 patients (80.9%) underwent testing with IGRA: 15 patients (1.2%) tested positive, 21 patients (1.7%) had invalid or indeterminate results, and 1,222 patients (97.1%) tested negative. Of those who had positive or invalid/indeterminate IGRA tests, 22 patients (61.1%) had tests that did not adhere to current guidelines.

**Conclusion:**

Nearly two-thirds of PLHIV who had a positive or invalid/indeterminate IGRA test during LTBI screening received testing that was not indicated under current guidelines. Over-testing may increase false-positive results, which can further lead to unnecessary treatment and drive healthcare costs. Targeted interventions, including clinician education and system-based changes such as EMR-based support tools, warrant further exploration to improve guideline adherence.

**Disclosures:**

All Authors: No reported disclosures

